# A novel de novo dominant mutation in *ISCU* associated with mitochondrial myopathy

**DOI:** 10.1136/jmedgenet-2017-104822

**Published:** 2017-10-27

**Authors:** Andrea Legati, Aurelio Reyes, Camilla Ceccatelli Berti, Oliver Stehling, Silvia Marchet, Costanza Lamperti, Alberto Ferrari, Alan J Robinson, Ulrich Mühlenhoff, Roland Lill, Massimo Zeviani, Paola Goffrini, Daniele Ghezzi

**Affiliations:** 1 Molecular Neurogenetics Unit, Foundation IRCCS Neurological Institute Besta, Milan, Italy; 2 Medical Research Council Mitochondrial Biology Unit, University of Cambridge, Cambridge, UK; 3 Department of Chemistry, Life Sciences and Environmental Sustainability, University of Parma, Parma, Italy; 4 Department of Medicine, Institut für Zytobiologie und Zytopathologie, Philipps-Universität, Marburg, Germany; 5 Unit of Metabolism, LOEWE Zentrum für Synthetische Mikrobiologie SynMikro, Marburg, Germany

**Keywords:** Iscu, Mitochondrial Myopathy, De Novo Mutation, Fe-s Cluster

## Abstract

**Background:**

Hereditary myopathy with lactic acidosis and myopathy with deficiency of succinate dehydrogenase and aconitase are variants of a recessive disorder characterised by childhood-onset early fatigue, dyspnoea and palpitations on trivial exercise. The disease is non-progressive, but life-threatening episodes of widespread weakness, metabolic acidosis and rhabdomyolysis may occur. So far, this disease has been molecularly defined only in Swedish patients, all homozygous for a deep intronic splicing affecting mutation in *ISCU* encoding a scaffold protein for the assembly of iron–sulfur (Fe-S) clusters. A single Scandinavian family was identified with a different mutation, a missense change in compound heterozygosity with the common intronic mutation. The aim of the study was to identify the genetic defect in our proband.

**Methods:**

A next-generation sequencing (NGS) approach was carried out on an Italian male who presented in childhood with ptosis, severe muscle weakness and exercise intolerance. His disease was slowly progressive, with partial recovery between episodes. Patient’s specimens and yeast models were investigated.

**Results:**

Histochemical and biochemical analyses on muscle biopsy showed multiple defects affecting mitochondrial respiratory chain complexes. We identified a single heterozygous mutation p.Gly96Val in *ISCU*, which was absent in DNA from his parents indicating a possible de novo dominant effect in the patient. Patient fibroblasts showed normal levels of ISCU protein and a few variably affected Fe-S cluster-dependent enzymes. Yeast studies confirmed both pathogenicity and dominance of the identified missense mutation.

**Conclusion:**

We describe the first heterozygous dominant mutation in *ISCU* which results in a phenotype reminiscent of the recessive disease previously reported.

## Introduction

Iron–sulfur (Fe-S) clusters are prosthetic groups found in several mitochondrial, cytosolic and nuclear enzymes, which play a role in fundamental cellular processes, such as respiration, DNA synthesis and repair, ribosome biogenesis and iron metabolism. In eukaryotes, the biogenesis of Fe-S clusters is performed by two main multiprotein machineries, the ISC (iron–sulfur cluster assembly) machinery localised in mitochondria and the CIA (cytosolic iron–sulfur protein assembly) machinery localised in the cytosol.^1 2^ Fe-S clusters are found in almost all living organisms, and the most common stoichiometric species include [2Fe-2S], [3Fe-4S] and [4Fe-4S] structures in which the Fe ions are co-ordinated, for example, by cysteine thiol groups or histidine residues.

The ISC machinery is involved in the biogenesis of Fe-S proteins in mitochondria enzymes as well as in the cytosol and nucleus. The current understanding of the molecular mechanism of mitochondrial Fe-S protein biogenesis has been worked out in both yeast and human cells. The ISC machinery comprises 18 known proteins that perform several steps of Fe-S cluster synthesis, transfer and insertion into recipient proteins including subunits of the mitochondrial respiratory chain (MRC) complexes I, II and III, mitochondrial aconitase (mACO) and lipoic acid synthase (LIAS).^3^ De novo synthesis of the [2Fe-2S] cluster is accomplished on the scaffold protein ISCU. This reaction requires the cysteine desulfurase NFS1 with its stabilising partners ISD11/LYRM4 and ACP1, frataxin/FXN as an iron donor and/or regulator of cysteine desulfurase activity, and the ferredoxin FDX2 (MIM 614585) as an electron donor for sulfur reduction.^4 5^ Conflicting results have been published regarding the role of FDX1 (MIM 103260) in this process.^6 7^ All these ISC proteins form a dynamic complex with ISCU. Dissociation of the preformed Fe-S cluster from the ISCU scaffold and transfer to intermediate carriers, for example the monothiol glutaredoxin GLRX5 (MIM 609588), is mediated by a dedicated Hsp70-Hsp40 chaperone system (HSC20/HSP70).^8^ The transiently GLRX5-bound [2Fe-2S] cluster is inserted into [2Fe-2S] targets or used for [4Fe-4S] cluster synthesis by ISCA1-ISCA2-IBA57. Finally, the cluster is inserted into target apoproteins with the help of factors including IND1 (MIM 613621), NFU1 (MIM 608100) and BOLA3 (MIM 613183). A different targeting mode has been suggested, based on interactions of the adaptable HSC20/HSPA9 scaffold complex with LYR motifs of SDHAF1 for complex II,^9^ with LYRM7 for complex III or directly with Fe-S cluster subunits of complex I.^10^

Recessive mutations in *ISCU* have been described in patients presenting myopathy with severe exercise intolerance and myoglobinuria (MIM 255125). A homozygous intronic transversion (c.418+382G>C or IVS5 +382G>C) was initially reported in patients from northern Sweden, with associated deficiencies of succinate dehydrogenase and aconitase in skeletal muscle.^11 12^ The mutation causes the retention of an ‘extra exon’, leading to marked reduction of *ISCU* mRNA and protein in patient muscle. The splicing defect was shown to be selective for muscle tissue, thus explaining the muscle-specific phenotype of this disorder.^13^ Later, compound heterozygosity for the common intronic mutation and a missense c.149G>A/p.G50E substitution was found in two brothers with Swedish/Finnish origin. These boys had a more severe phenotype than patients homozygous for the intronic mutation, with progressive and severe muscle weakness, muscle wasting and heart involvement.^14^

Contrary to the muscular phenotype of *ISCU* mutant patients, mutations in other components of the core Fe-S assembly complex cause neurological diseases (eg, Friedreich’s ataxia, MIM 229300, due to *FXN* mutations) or multisystem disorders (eg, combined oxidative phosphorylation deficiency 19, MIM 615595, due to *ISD11/LYRM4* mutations).

We report here a patient with myopathy, lactic acidosis and combined MRC complex deficiency, caused by a de novo heterozygous missense pathological variant in *ISCU*.

## Methods

### Histochemical and biochemical studies in skeletal muscle

Muscle morphology and histochemistry, respiratory chain activities of complexes I to IV and pyruvate dehydrogenase complex (PDHC) assays were performed as previously described.^15–17^ Histochemical staining of iron using Prussian blue colour was performed as previously described.^18^

### Genetic analysis

Genomic DNA was extracted from peripheral blood by standard methods. Whole exome sequencing (WES) and variants filtering were performed as previously described.^19^ Variants identified by WES were validated by Sanger sequencing. For deep sequencing of parental DNAs, the PCR products were processed with Nextera XT DNA sample preparation kit (Illumina). Sequencing was performed on an Illumina MiSeq instrument.

RNA was extracted from skin fibroblasts, and 1 µg was used as template for RT-PCR to obtain full-length cDNA. *ISCU* transcript was amplified by PCR and run through a 1% agarose gel in order to detect potential splicing alterations. PCR products were also sequenced in order to confirm genomic variants and unmask potential events of nonsense-mediated decay.

### Cell culture and biochemical analysis of fibroblast samples

Fibroblasts obtained from skin biopsy were grown in 1 g/L glucose DMEM-F14 (Euroclone) supplemented with 20% fetal bovine serum (FBS), 1% uridine, 1% L-glutamine and 0.2% sodium pyruvate.

For enzyme activity measurements, cells were treated with digitonin in order to separate a cytosolic cell fraction from a crude mitochondria-containing organellar fraction.^20^ Biochemical assays were essentially performed as described.^16^ Analysis of steady-state protein levels by immunoblotting was carried out by common methods, using a 6%–20% sodium dodecyl sulfate polyacrylamide (SDS-PA) gradient gel. Blocked membranes were probed with primary antibodies (online supplementary table S1), and antigens were visualised by horseradish peroxidase (HRP) coupled secondary reagents and a chemiluminescence reaction.

10.1136/jmedgenet-2017-104822.supp1 Supplementary material 1

### Yeast studies

Details on yeast strains, media, cloning procedures and vectors^21 22^ as well as on generation of mutant allele and construction of mutant strains^23–25^ are reported in the online supplementary data

Complex II (succinate dehydrogenase (SDH)) and complex IV (cytochrome c oxidase (COX)) specific activities were measured on a mitochondrial-enriched fraction prepared as previously described.^26 27^ Aconitase activity was measured in whole-cell extracts.^28^ In vivo radiolabelling of yeast cells with ^55^FeCl_3_ (ICN) and measurement of ^55^Fe-incorporation into Fe-S proteins by immunoprecipitation and scintillation counting were performed as described.^29^ Antibodies against c-Myc were obtained from Santa-Cruz. The green fluorescent protein (GFP) based reporter assay for determination of *FET3* promoter strength was described previously.^29^ The iron content was determined by a colorimetric assay, essentially as described before.^30 31^

### Bioinformatics and structural analysis tools

The effect of the p.(Gly96Val) substitution on ISCU function was predicted using the in silico tools (ie, SIFT, Polyphen2 and EVmutation), all running recommended parameters. All images of the *Escherichia coli* IscU-IscS (PDB ID:3LVL) complex were made by using Visual Molecular Dynamics viewer.^32^ The evaluation of the impact of the mutated residue and the protein–protein interaction analysis were performed by using Swiss PDB viewer,^33^ and FirstGlance in Jmol (http://www.jmol.org)

## Results

### Case report

The patient is a 23-year-old Italian male, first child from non-consanguineous parents, born at term after a normal pregnancy by caesarean delivery. He has a healthy younger brother. He started walking at 18 months, but he always presented some walking difficulties, with frequent falls. Parents reported easy fatigability since the first years of life. At 7 years of age, the neurological examination showed bilateral ptosis not associated with ophthalmoparesis, muscle hypotonia and wasting, and absent deep tendon reflexes. No obvious weakness was present, but marked exercise intolerance was reported. Neither cognitive impairment nor other central nervous system (CNS) involvements were noticed. The brain nuclear magnetic resonance (NMR) and the EEG were normal. Electromyography showed myopathic changes in all tested muscles. Creatine kinase (CK) level was slightly increased (about 300 U/L; normal values, n.v.:<180), lactate acid was high in blood (4.6 mmol/L; n.v.: 0.4–2.2). He showed leucopenia (2.94×10^3^/µL; n.v.: 5–14) and anaemia (haemoglobin 10.9 g/dL; n.v. 13–16) with microcythemia (72.9; n.v. 80–99); a bone marrow biopsy performed at 7 years was normal. No heart involvement was present: echocardiogram and ECG were both normal. Over time, his clinical conditions worsened and he started presenting episodes of profound exercise intolerance and weakness, with partial recovery of muscle weakness between episodes in about 2 weeks. During these episodes, the patient was unable to walk, and showed tachycardia; neither breathing shortness nor dysphagia was noticed. At the neurological examination at 17 years of age, he presented predominantly distal limb weakness with muscle hypotrophy; deep tendon reflexes were absent. He was able to stand up from a chair and walk, but was unable to run. No signs of CNS involvement were present; CK level remained high (about 1700 U/L). The disease has since been slowly progressive, punctuated by episodes of acute weakness, with preserved cognitive function and no other signs of CNS involvement.

### Histochemical and biochemical analyses in skeletal muscle

**Figure 1:**
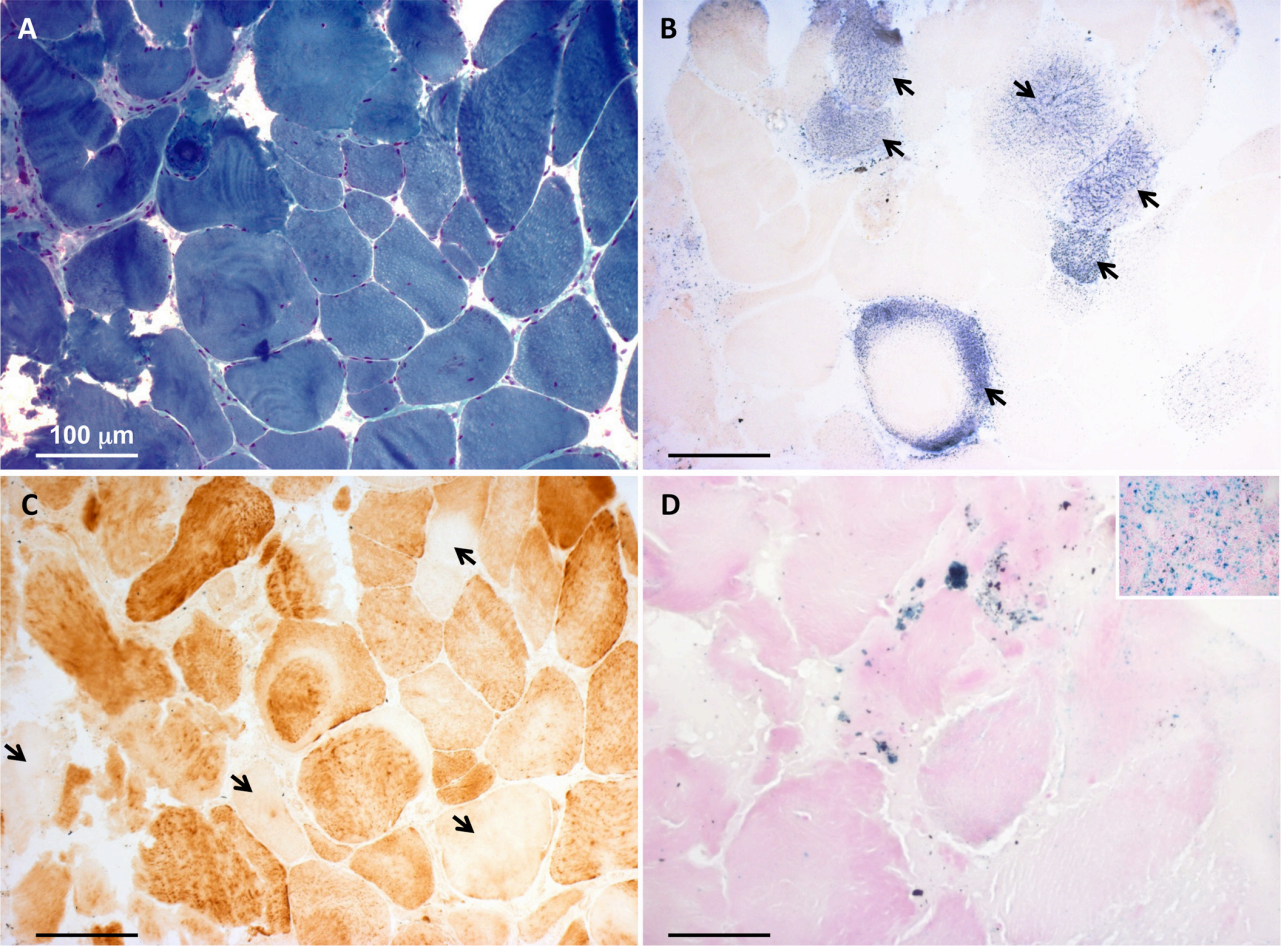
Histochemical analysis of the patient’s muscle biopsy. (A) Gomori trichrome stain showing fibre size variability. (B) Strongly reduced histochemical activity of succinate dehydrogenase. Few fibres showed succinate dehydrogenase-positive staining (arrows). (C) Cytochrome c oxidase (COX) staining showing scattered fibres with severe reduction of histochemical COX activity (arrows). (D) Perls staining demonstrating punctuate accumulation of iron in the patient’s muscle fibres. Inset is a positive control (spleen) for the Prussian blue reaction. Bars correspond to 100 µm.

A first muscle biopsy was performed at 8 years of age. At the histological examination, fibre size variability was present. The main feature was a severe reduction of the histochemical reaction for both COX and SDH, not associated with ragged red fibres. The biochemical examination showed severe decrease of all the MRC complexes (I, II, III, IV), with strong increase in citrate synthase (CS) activity (online supplementary figure S1A). A second muscle biopsy was performed at 22 years, confirming the histological and histochemical findings (figure 1A-C). At this age, the reduction in the MRC complex activities was still present, yet normal CS activity was observed (online supplementary figure S1B). Furthermore, the activity of PDHC was reduced (PDH/CS: 1.8, normal values: 2.5–5.0). After the genetic identification of the *ISCU* variant (see below), we carried out histological analysis for the presence of iron deposits in the muscle biopsy by Perls staining. We found Perls-positive material in numerous patient’s fibres, whereas no such material was detected in control muscle biopsies, indicating iron overload in ISCU-mutant muscle (figure 1D).

### Genetic studies

**Figure 2:**
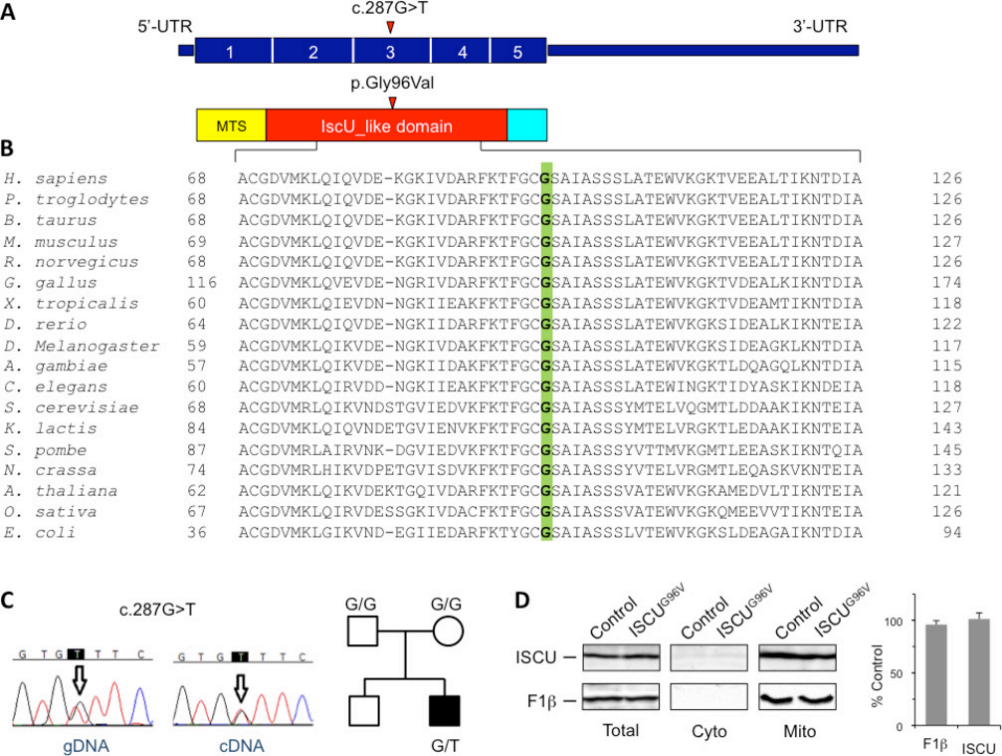
Identification and characterisation of an *ISCU* mutation. (A) Schematic representation of the *ISCU* cDNA (NM_213595.2) and ISCU protein with the nucleotide/amino acid change identified in this study. The functional IscU-like domain is in red; the mitochondrial targeting sequence (MTS) is in yellow. (B) Phylogenetic conservation of the amino acid residue (Gly96, in green) affected by the missense mutation identified in the patient. (C) Electropherograms of the genomic region (gDNA) and transcript (cDNA) harbouring the *ISCU* mutation, and pedigree. (D) Immunoblot analysis of ISCU^G96V^ mutant protein expression and subcellular localisation. Control and patient fibroblasts were harvested by trypsination, permeabilised by digitonin treatment, and separated into a cytosolic and a mitochondria-containing membrane fraction. Total cell lysates as well as cytosolic and crude mitochondrial fractions were subjected to sodium dodecyl sulfate polyacrylamide gel electrophoresis (SDS-PAGE) and analysed for ISCU and ATP synthase F1β subunit (mitochondrial marker) steady-state protein levels (left panel). Chemiluminescence signals of ISCU and F1β in total lysate samples were quantified, and values obtained from patient fibroblasts were expressed relative to control cells (right panel). Error bars indicate the SDs (n=3). UTR, untranslated region.

Genetic alterations linked to mitochondrial DNA were ruled out: no mutation was detected by sequencing and no evidence of depletion or deletion was observed by Southern blot analysis of mitochondrial DNA from muscle. We performed WES on genomic DNA from the proband. After filtering steps to exclude common SNPs (frequency >0.5%), we selected genes with two compound heterozygous or one homozygous variant, according to a predicted recessive mode of inheritance. Then we focused on genes encoding proteins with mitochondrial localisation. Two entries were found: *MTIF2* (translation initiation factor IF-2, mitochondrial) and *PDPR* (pyruvate dehydrogenase phosphatase regulatory subunit). However, the two missense variants in *MTIF2* were in cis, on the same paternal allele, whereas the two variants in *PDPR* were not confirmed by Sanger sequencing, being probably due to the presence of a pseudogene. No hemizygous variant, suggestive of an X-linked transmission, was detected. A deep analysis of the heterozygous variants, prioritising genes associated with mitochondrial myopathies, highlighted a single variant in *ISCU,* a c.287G>T (NM_213595.2) predicted to cause the amino acid substitution p.G96V (figure 2A). This nucleotide change was not reported in public variant databases (dbSNP, EVS, ExAC (August 2016)); the substitution affected a highly conserved residue (figure 2B) and gave high scores of pathogenicity, according to several bioinformatics tools. This variant was confirmed by Sanger sequencing in the proband but was not present in the parents’ blood DNA, indicating a de novo event (figure 2C, online supplementary figure S2): the parental DNA samples were analysed also by deep sequencing to exclude very low level of the variant, suggestive of germinal mosaicism. All the *ISCU* coding regions were well covered by WES. We then screened our patient and his parents for the intronic region encompassing the common mutation present in all the previously described *ISCU* mutant patients but no variant was identified.

To exclude that WES could have missed the presence of another deep intronic variant affecting the splicing or a ‘non-exonic’ variant impairing mRNA transcription/stability, we further investigated patient’s specimen at the transcriptional level. No aberrant mRNA *ISCU* species was observed in PCR products obtained from fibroblast RNA, and their sequencing showed a biallelic expression, suggested by the presence of overlapping peaks corresponding to G and T nucleotides in position c.287 (online supplementary figure S2).

### Characterisation of patient’s fibroblasts

The amount of ISCU protein in patient’s fibroblasts was similar to controls, indicating that the mutant protein is normally synthesised, imported into mitochondria and stable (figure 2D). In line with previous reports showing that the biochemical phenotype associated with mutant ISCU is very much attenuated, fibroblasts harbouring the *ISCU* ^G96V^ mutation showed hardly any defect in activities or protein amounts for a number of mitochondrial Fe-S dependent enzymes including mACO, LIAS (as indicated by the presence of lipoate cofactor (Lip) on pyruvate and ketoglutarate dehydrogenase E2 subunits), ferrochelatase and respiratory chain complexes I, II and III (online supplementary figure S3A–F). A minor defect was observed in complex IV, in line with the muscle biopsy analyses, and in the activity of cytosolic aconitase (IRP1). Analysis of steady-state levels of cytosolic/nuclear Fe-S proteins as a measure for maturation-dependent stability revealed no general alteration of their cellular abundance (online supplementary figure S3G-H). However, we observed a severe deficiency of the base-excision DNA repair enzyme NTHL1, a [4Fe-4S] protein, and a slight decrease in protein levels of the CIA factor IOP1 containing 2 [4Fe-4S] clusters. Taken together, our analyses indicate that the presence of the *ISCU* ^G96V^ mutation does not have a strong impact on Fe-S cluster assembly in patient’s cultured fibroblasts.

### Yeast model

To assess the pathogenic role of the substitution p.Gly96Val identified in the patient, we performed studies in a yeast model, by introducing the analogous amino acid substitution (G97V) in the *S*accharomyces* cerevisiae* orthologue of *ISCU*, the yeast gene *ISU1. ISU1* has a paralogue, *ISU2*, arising from a recent gene duplication. The double deletion mutant *isu1*Δ*isu2*Δ is unviable, thus indicating the essential role of these proteins in the biogenesis of Fe-S clusters, which is in turn indispensable for yeast cell survival.^34^ The double deletion mutant *isu1*Δ*isu2*Δ, harbouring the centromeric pFL38 plasmid (*URA3* marker) with the wild-type (wt) *ISU1* to allow viability, was additionally transformed with pFL39 centromeric plasmids (*TRP1* marker) containing either the mutant allele *isu1^G97V^*, a wt copy of *ISU1,* or no gene.

**Figure 3:**
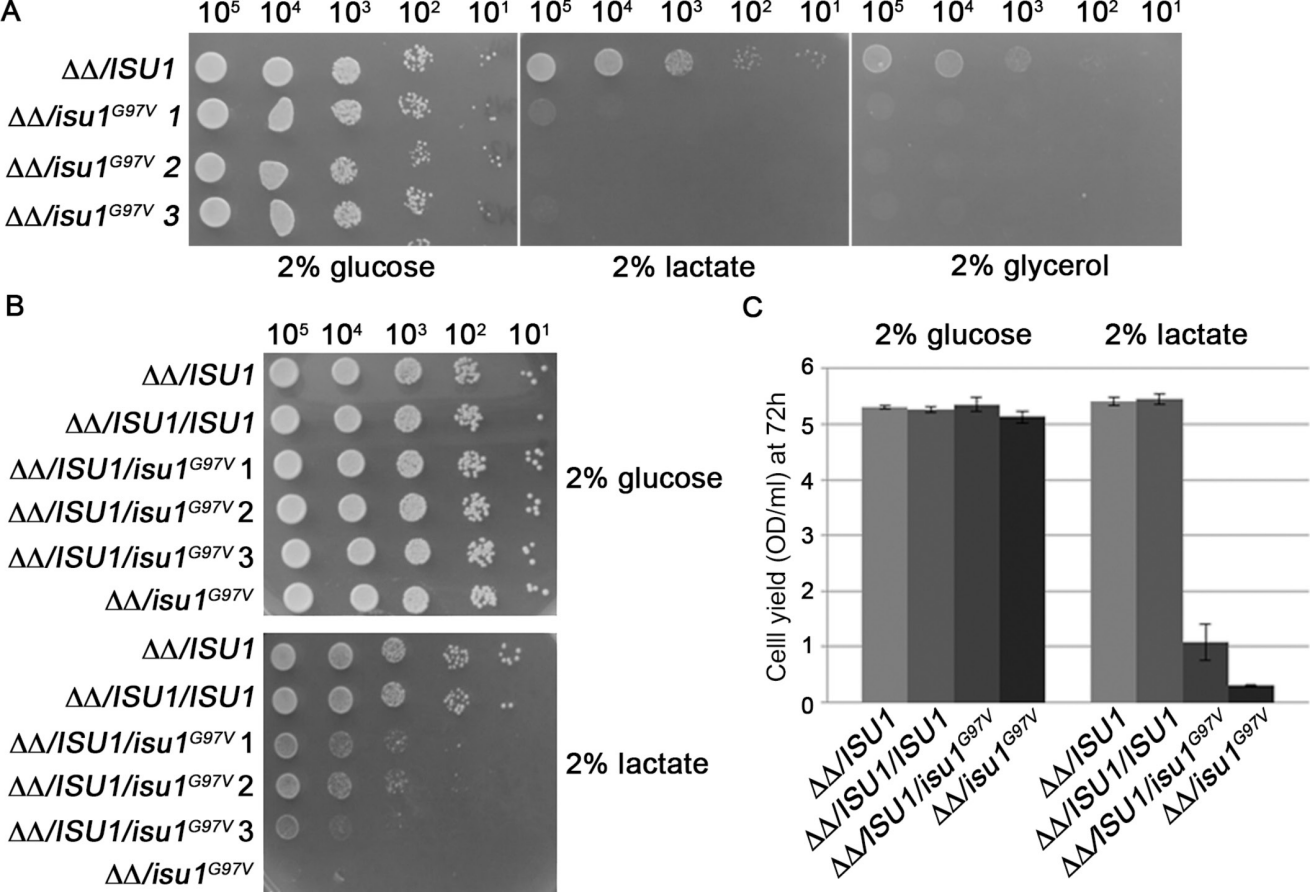
Growth analysis of mutant yeast strains. (A) The strain *isu1*Δ*isu2*Δ harbouring plasmid pFL39 with the wild-type *ISU1* gene or the mutant allele *isu1* ^G97V^ was analysed for growth on various media. Equal amounts of serial dilutions of cells from exponentially grown cultures were spotted onto yeast nitrogen base (YNB) medium plus 2% glucose, 2% lactate or 2% glycerol. The growth was scored after 3 days of incubation at 28°C. (B) The strains *isu1*Δ*isu2*Δ/*ISU1* and *isu1*Δ*isu2*Δ/*isu1* ^G97V^ were transformed with pFL38/ISU1 or with the empty vector. Equal amounts of serial dilutions of cells from exponentially grown cultures were analysed for growth on YNB medium plus 2% glucose or 2% lactate after 4 days of incubation at 28°C. (C) Cell yield was calculated by growing cells on liquid medium containing glucose or lactate and measuring the optical density at 600 nm after 72 hours of growth. Error bars indicate the SDs (n=3).

The different strains were plated on 5-fluoroorotic acid containing medium to select for cells that have lost the pFL38/*ISU1* plasmid. The strain expressing *isu1^G97V^* as the sole *ISU1* gene was able to grow on glucose at rates similar to strains carrying the wt *ISU1*, while the empty pFL39 did not support growth (figure 3A). This result indicates that glycine 97 is not essential for the function of the Isu1 protein. However, growth of the strain expressing the *isu1^G97V^* variant was severely retarded on non-fermentable carbon sources (figure 3A), highlighting a deleterious effect of the G97V mutation on mitochondrial function.

To test whether the G97V mutation acts as a dominant trait, the *isu1*Δ*isu2*Δ/*isu1^G97V^* and the *isu1*Δ*isu2*Δ/*ISU1* strains were transformed with either the pGL38 empty vector (as a control) or pFL38/*ISU1* thus obtaining the heteroallelic strain *isu1*Δ*isu2*Δ/*ISU1*/*isu1^G97V^* and the homoallelic strain *isu1*Δ*isu2*Δ/*ISU1*/*ISU1*. Growth on non-fermentable carbon sources was clearly reduced in the heteroallelic strain compared with the homoallelic wt strain, and also to the strain transformed with the empty vector and expressing a single copy of *ISU1 (isu1*Δ*isu2*Δ/*ISU1)*. These results indicate that the G97V mutation behaves as dominant (figure 3B). The measurement of the cell yield in liquid cultures confirmed what observed in the spot assay analysis (figure 3C).

**Figure 4:**
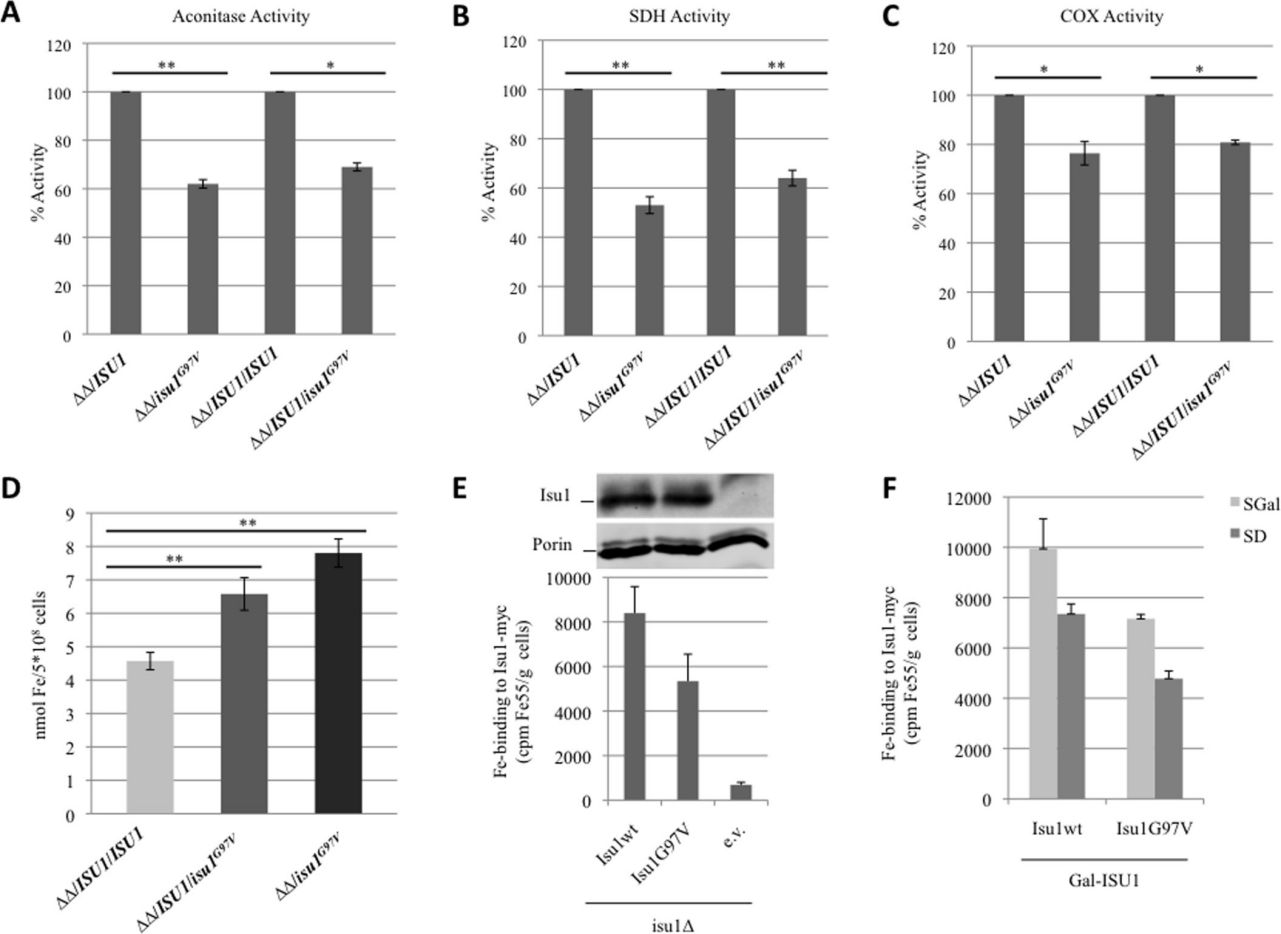
Measurement of enzyme activities and iron content in yeast. (A) Aconitase activity was measured in whole-cell extracts from cells grown exponentially at 28°C in yeast nitrogen base (YNB) medium plus 0.6% glucose. (B and C) Succinate dehydrogenase activity and cytocrome c oxidase activities were measured in a mitochondria-enriched fraction obtained from cells grown as described before. The values for *isu1*Δ*isu2*Δ/*isu1* ^G97V^ and *isu1*Δ*isu2*Δ*/ISU1*/*isu1* ^G97V^ strains are expressed as percentage of the activities obtained in the strains *isu1*Δ*isu2*Δ*/ISU1* and *isu1*Δ*isu2*Δ*/ISU1*/*ISU1.* (D) Cellular iron content was quantified in cells grown up to early stationary phase in YNB 0.2% glucose and 2% galactose medium. *<0.05 (unpaired two-tailed t-test), **<0.01 (unpaired two-tailed t-test). (E) *Gal-ISU1/isu2Δ* cells and *isu1Δ* cells expressing Myc-tagged Isu1 were radiolabelled with ^55^Fe and ^55^Fe incorporation into Isu1-Myc was determined by immunoprecipitation with α-Myc antibodies followed by scintillation counting. Wild-type cells harbouring the empty vector (e.v.) served as control. Isu1-myc protein levels in *isu1Δ* cells were determined by immunostaining with α-Myc antibodies. Porin (Por1) served as a loading control. (F) *Gal-ISU1/isu2Δ* cells expressing Isu1 from vector pFL39 and the reporter plasmid pFET3-GFP were cultivated in SD or SGal medium supplemented with 50 µM ferric ammonium citrate. At an optical density=0.5, the GFP-specific fluorescence emission of whole cells was determined. Error bars indicate the SDs (n=3).

To investigate if the G97V mutation affects mitochondrial Fe-S protein biogenesis, we measured the biochemical activities of two Fe-S cluster-containing enzymes: aconitase and complex II. These activities were reduced in both the *isu1*Δ*isu2*Δ/*isu1^G97V^* and the heteroallelic strain *isu1*Δ*isu2*Δ/*ISU1*/*isu1^G97V^* (figure 4A and B) indicating an impairment in Fe-S cluster biogenesis in *isu1^G97V^*-containing yeast cells. The activity of complex IV which contains two heme centres was also partially reduced (figure 4C). Finally, we evaluated the cellular iron content by a colorimetric assay and found a twofold increase in the mutant compared with wt strain (figure 4D) consistent with a defective core ISC machinery.^35^

To further investigate the impact of the G97V mutation on Isu1 function, we measured the ability of mutant versus wt Isu1 protein to assemble a Fe-S cluster in vivo.^36^ To this end, we used the strain *Gal-ISU1/isu2Δ,* in which *ISU2* is deleted and *ISU1* is under the control of the *GAL1-10* promoter. The levels of Isu1 can be downregulated by growing the cells in the presence of glucose.^37^ *Gal-ISU1/isu2Δ* cells were transformed with pRS426-TDH3 vectors encoding Myc-tagged *ISU1^wt^* or *isu1^G97V^*. To estimate Fe-S cluster binding of Isu1, cells were radiolabelled with ^55^Fe, and the incorporation of radioactivity into Isu1^G97V^ or Isu1^wt^ was measured by immunoprecipitation and scintillation counting (figure 4E,F). As shown in figure 4F (darker bars), the mutant protein showed a 30% reduction in the ^55^Fe binding capacity; this reduction is consistent with the mutant growth defect displayed on respiratory carbon sources. A decrease was also evident when *isu1^G97V^* was expressed in *isu1Δ* cells or when *Gal-ISU1/isu2Δ* cells were depleted by growth in galactose-containing medium, conditions in which endogenous wt Isu1 and Isu2 are present, thus mimicking the heteroallelic condition (figure 4F, light grey bars, and figure 4E). This result showed that the Isu1^G97V^ mutant protein is slightly impaired in its ability to incorporate Fe into Fe-S clusters in vivo. The alterations observed in mutant strains were not due to a reduced amount of the mutant Isu1^G97V^ protein since its abundance was similar to the wt form (figure 4E). Furthermore, *Gal-ISU1/isu2Δ* cells expressing *isu1^G97V^* from vector pFL39 displayed a twofold increase of the iron-dependent *FET3* promoter on both glucose and galactose-containing medium (online supplementary figure S4). A deregulated iron homeostasis is a hallmark of cells with defective core ISC machinery and explains the increased iron levels in cells expressing *isu1^G97V^*.

### *In silico* pathogenicity prediction and structural modelling

**Figure 5:**
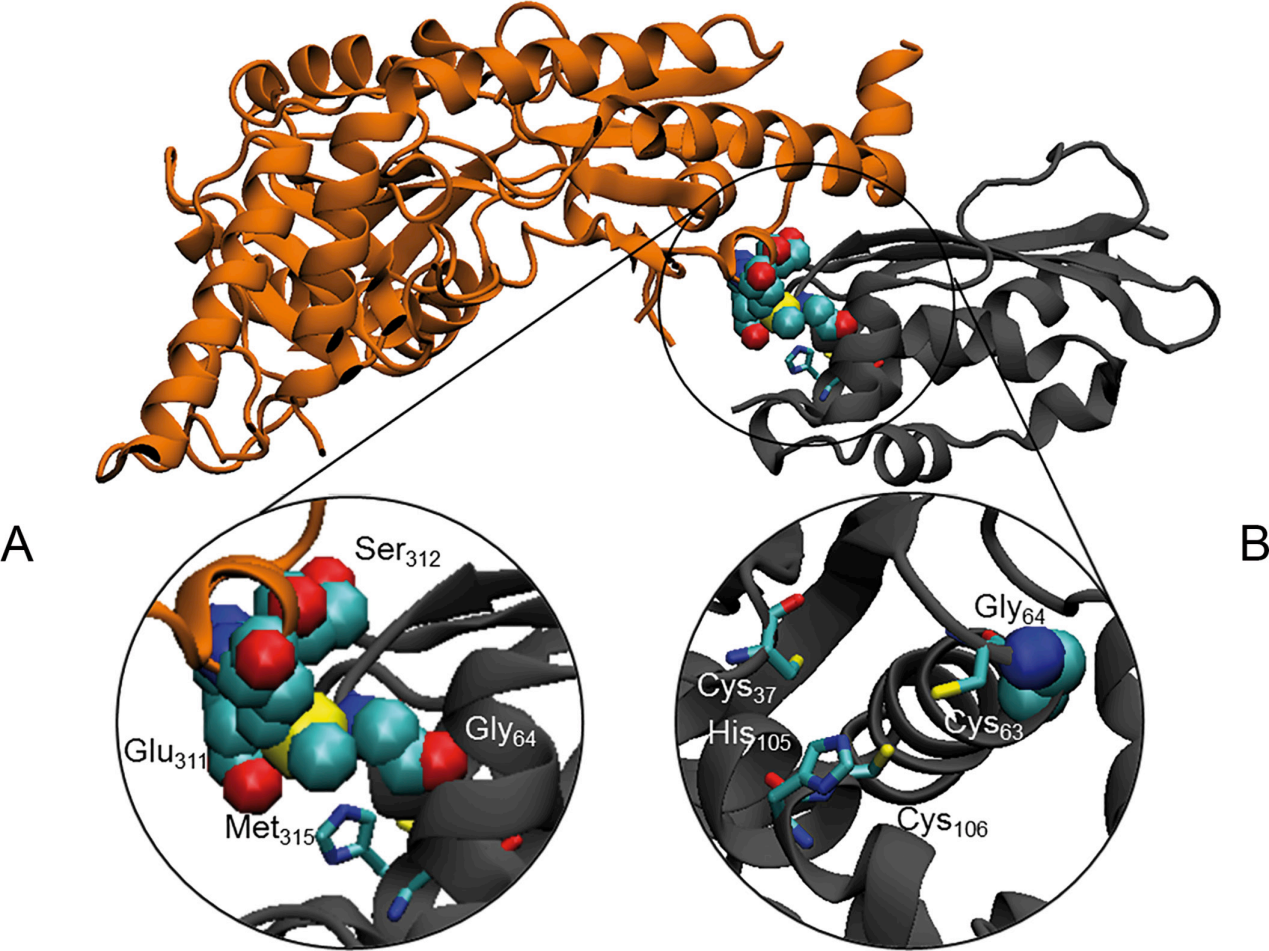
In silico structural analysis. (A) Ribbon diagram of the *E*scherichia* coli* IscS (coloured in grey)-IscU (coloured in orange) complex (PDB ID: 3lvl). Close view of the Gly64 of IscU and Glu311, Ser312 and Met315 of IscS represented in Van der Waals and coloured by type. (B) Residues supposed to be involved in the co-ordination of the 2Fe-2S are represented as sticks.

In silico predictions, by using different tools, were used to evaluate the potential effect of the Gly96Val substitution on ISCU structure and interaction with its protein partners. ISCU interacts with NFS1, yet the 3D structure of the human NFS1-ISCU complex is hitherto unknown. Instead, we used the reported crystal structure of the *E. coli* IscS-IscU complex formed by an IscS dimer which binds two IscU at opposite ends of IscS.^38^ Notably a similar, yet unusual monomeric structure has been recently described for the IscS human orthologue NFS1.^39^ Human Gly_96_ which corresponds to Gly_64_ in *E. coli* 38 is located in a flexible loop, at the beginning of the helix α1, next to the potential Fe-S cluster-co-ordinating Cys_95_ residue (Cys_63_ in *E. coli*).^40^ We analysed the possible interactions of Gly_64_ with IscS/NFS1 by using First Glance in Jmol. The glycine backbone could establish hydrophobic interaction with IscS Met_315_ that is located inside a stretch (YVEGESLLMAL) highly conserved from *E. coli* to human. Although both glycine and valine are hydrophobic residues, the greater steric hindrance of the valine side chain could interfere with the IscS-IscU interaction (figure 5A). Moreover, the loop encompassing residues 62–64 (GCG) of IscU is opposite to Glu_311_ and Ser_312_ on IscS, and we suggest that the valine slightly disturbs these hydrophilic residues (figure 5A). The substitution Gly96Val could therefore prevent the correct orientation of the adjacent cysteine side chain, thus interfering with the Fe co-ordination and explaining the defective ISCU function (figure 5B).

## Discussion

ISCU (Isu1-2 in yeast) is an essential scaffold protein for the biosynthesis of Fe-S clusters. Recessive hypomorphic *ISCU* alleles have been associated with isolated myopathy^11 12^ or skeletal and cardiac myopathy^14^ in humans. Here, we report a heterozygous, de novo dominant *ISCU* variant, G96V, causing congenital myopathy in a single patient. The following considerations provide evidence that this variant exerts a deleterious, dominant effect on Fe-S-dependent enzyme activities. First, we observed striking clinical, histological, histochemical and biochemical abnormalities affecting skeletal muscle, which define a mitochondrial myopathy similar to that reported in *ISCU* recessive mutations, including partial depletion of SDH and COX histochemical reactions, generalised reduction of the MRC complex activities, and accumulation of iron deposits. Similar to recessive *ISCU* cases, our patient displayed isolated myopathy with fluctuating, waxing and weaning episodes of profound muscle weakness, in the context of a congenital myopathy with moderately high CK and no involvement of extramuscular organs, including the CNS. Second, the mutation was absent in both healthy parents, supporting its sporadic occurrence in the proband, and was the only gene defect related to mitochondrial myopathies identified by WES. Third, analysis of the corresponding genetic defect in yeast suggested a Fe-S protein biogenesis defect, including defects in OXPHOS and the cellular iron regulon. The effects were dominant, since both the monoallelic and the heteroallelic genotypes were associated with the phenotype.

While our yeast studies suggest that Isu1^G97V^ is functionally impaired by itself, what might then be the reason for the observed dominance of both the human ISCU^G96V^ and corresponding yeast Isu1^G97V^ mutations? ISCU, as the scaffold protein for the de novo synthesis of Fe-S clusters within mitochondria, interacts with a number of other ISC proteins during Fe-S biosynthesis, although the stoichiometry of these interactions is controversial. Similar to the bacterial structure, the mammalian ISC complex was proposed to be composed of two NFS1 and two ISCU subunits,^4 41^ with a central NFS1 dimer and two molecules of ISCU at each end of the NFS1 dimer. Recently, a [FXN^42–210^]_24_-[NFS1]_24_-[ISD11]_24_-(ISCU)_24_ complex model was proposed,^40^ but it was obtained by overexpressing human proteins in *E. coli* and it is probably not relevant in vivo. We think that the functional impairment of ISCU by the G96V mutation may be caused by structural changes, as suggested by our in silico analyses. For instance, the orientation of the potential Fe-S cluster-co-ordinating Cys95 adjacent to Gly96 could be altered by replacement of the helix-breaking glycine residue, with a negative outcome on protein function in Fe-S cluster assembly. Moreover, the mutated protein structure may become stiffer, thus negatively affecting the interactions with its other partner ISC proteins, notably NFS1. The altered interaction between NFS1 and the mutant ISCU could in turn affect the assembly/function of the entire ISC biosynthetic complex. In support of this view stands the observation that the ISCU^G96V^ mutant protein is stable and not degraded, and its amount is similar to that of ISCU^wt^ in patient’s fibroblasts. In addition, the dominant effect may be caused by the intermediate formation of an ISCU holodimer which, according to in vitro reconstitution studies, is the product of the ISC biosynthetic complex.^5 42^ In the homodimer, the mutated Gly_96_ residue in one ISCU monomer would likely directly face the other wt ISCU monomer because of the bridging character of [2Fe-2S] cluster binding. This could lead to the observed dominant negative effect of the mutated ISCU on wt ISCU proteins. This might also be the reason why we observed less radiolabelled ^55^Fe bound to yeast Isu1^G97V^compared with the wt protein. Interestingly, the dominant-negative behaviour of the G97V mutation differs from the recessive effect reported for the G50E mutation in both yeast and humans, which can be ascribed to haploinsufficiency of the G50E allele.^43^

Although ubiquitous, the ISCU^G96V^ variant produces a clinically detectable effect only in one critical tissue, skeletal muscle, in line with the purely myopathic presentation of the common *ISCU^IVS5+382G>C^* splicing variant already reported in Swedish patients. Similar to our results, no evident mitochondrial phenotype in fibroblasts has been previously reported in cases with ISCU-related recessive disease.^14 44–46^ The molecular mechanisms that make skeletal muscle exquisitely sensitive to partial ISCU impairment warrant further investigation in patient-derived cells and, possibly, animal models. A tissue-specific splicing of *ISCU* was proposed for the common *ISCU^IVS5+382G>C^* mutation to explain the skeletal muscle phenotype,^13^ but this hypothesis cannot be applied for either the p.G96V or the p.G50E missense variants, which are equally associated with muscle-specific dysfunction. The structural alterations and impaired interactions with partner proteins caused by the p.G96V could have tissue-specific effects which explain the muscle involvement. A greater sensitivity of *ISCU*-mutant myoblasts to oxidative stress,^45^ and exercise-induced oxidative damages in muscle,^47^ may also account for the muscular phenotype. Nevertheless, tissue specificities for many of the mitochondrial diseases due to defective nuclear-encoded genes with housekeeping functions are currently poorly understood and hardly predictable.

The multiple biochemical defects of MRC activities observed in our patient were expected for complexes I, II and III which contain Fe-S clusters. A defect was also seen for the Fe-S cluster-free complex IV, which harbours Cu^2+^ and Fe-containing heme *a/a3* as redox centres. In principle, this may be due to a defect in the Fe-S protein ferrochelatase catalysing the last step of heme biosynthesis.^48^ However, strong complex IV defects are also seen on depletion of *ISCA1*, *ISCA2* and *IBA57* in both human and yeast cells.^49 50^ These ISC proteins are specific for mitochondrial [4Fe-4S] protein maturation, yet are not involved in the assembly of the [2Fe-2S] cluster on human ferrochelatase. Moreover, we observed a complex IV impairment in the mutant yeast model despite the yeast orthologue enzyme is devoid of a Fe-S cluster. Nevertheless, complex IV defects are common secondary effects of an impairment of Fe-S protein biogenesis.^6 49^ A complex IV deficiency (19%–28% residual activity) was also reported in the two siblings with the most severe *ISCU*-related phenotype, and a partial reduction was found in five subjects with the Swedish-type myopathy.^14^ This may be due to a downstream damaging effect linked to impairment of complexes I, II and III or to the hampered formation of MRC supercomplexes. In addition to the biochemical defects, the histochemical analysis was peculiar, with a quite specific pattern of SDH and COX deficiency; this was suggested to be a pathognomonic finding of a myopathy related to Fe-S cluster.^9^ However, in patients with mutations in *FXN*, the picture is different, with mainly COX negative fibres and nearly normal SDH staining.^51^ Other Fe-S diseases are rare and usually present as neurological disorders with minimal myopathic signs, while little is known about their muscle features. Collectively, the histochemical pattern in muscle biopsy seems specific for ISCU myopathy.

In conclusion, we report the first heterozygous dominant mutation in *ISCU*; notably, this alteration resulted in a similar phenotype as the recessive *ISCU* disease previously described. Our finding stresses the importance of a deep analysis of WES data that may include, for sporadic cases, any mode of transmission. Moreover, our study confirms that recessive and dominant mutations in the same gene may lead to the same disease, as already reported for other mitochondrial disorders (eg, *DNM1L* mutations).

## References

[1] Lill R, Hoffmann B, Molik S, Pierik AJ, Rietzschel N, Stehling O, Uzarska MA, Webert H, Wilbrecht C, Mühlenhoff U The role of mitochondria in cellular iron-sulfur protein biogenesis and iron metabolism. Biochim Biophys Acta 2012;1823:1491–508. 10.1016/j.bbamcr.2012.05.009 22609301

[2] Rouault TA Biogenesis of iron-sulfur clusters in mammalian cells: new insights and relevance to human disease. Dis Model Mech 2012;5:155–64. 10.1242/dmm.009019 22382365 PMC3291637

[3] Stehling O, Lill R The role of mitochondria in cellular iron-sulfur protein biogenesis: mechanisms, connected processes, and diseases. Cold Spring Harb Perspect Biol 2013;5:a011312 10.1101/cshperspect.a011312 23906713 PMC3721283

[4] Schmucker S, Martelli A, Colin F, Page A, Wattenhofer-Donzé M, Reutenauer L, Puccio H Mammalian frataxin: an essential function for cellular viability through an interaction with a preformed ISCU/NFS1/ISD11 iron-sulfur assembly complex. PLoS One 2011;6:e16199 10.1371/journal.pone.0016199 21298097 PMC3027643

[5] Webert H, Freibert SA, Gallo A, Heidenreich T, Linne U, Amlacher S, Hurt E, Mühlenhoff U, Banci L, Lill R Functional reconstitution of mitochondrial Fe/S cluster synthesis on Isu1 reveals the involvement of ferredoxin. Nat Commun 2014;5:5013 10.1038/ncomms6013 25358379

[6] Sheftel AD, Stehling O, Pierik AJ, Elsässer HP, Mühlenhoff U, Webert H, Hobler A, Hannemann F, Bernhardt R, Lill R Humans possess two mitochondrial ferredoxins, Fdx1 and Fdx2, with distinct roles in steroidogenesis, heme, and Fe/S cluster biosynthesis. Proc Natl Acad Sci U S A 2010;107:11775–80. 10.1073/pnas.1004250107 20547883 PMC2900682

[7] Shi Y, Ghosh M, Kovtunovych G, Crooks DR, Rouault TA Both human ferredoxins 1 and 2 and ferredoxin reductase are important for iron-sulfur cluster biogenesis. Biochim Biophys Acta 2012;1823:484–92. 10.1016/j.bbamcr.2011.11.002 22101253 PMC3546607

[8] Ye H, Jeong SY, Ghosh MC, Kovtunovych G, Silvestri L, Ortillo D, Uchida N, Tisdale J, Camaschella C, Rouault TA Glutaredoxin 5 deficiency causes sideroblastic anemia by specifically impairing heme biosynthesis and depleting cytosolic iron in human erythroblasts. J Clin Invest 2010;120:1749–61. 10.1172/JCI40372 20364084 PMC2860907

[9] Maio N, Ghezzi D, Verrigni D, Rizza T, Bertini E, Martinelli D, Zeviani M, Singh A, Carrozzo R, Rouault TA Disease-Causing SDHAF1 Mutations Impair Transfer of Fe-S Clusters to SDHB. Cell Metab 2016;23:292–302. 10.1016/j.cmet.2015.12.005 26749241 PMC4749439

[10] Maio N, Kim KS, Singh A, Rouault TA A Single Adaptable Cochaperone-Scaffold Complex Delivers Nascent Iron-Sulfur Clusters to Mammalian Respiratory Chain Complexes I-III. Cell Metab 2017;25:945–53. 10.1016/j.cmet.2017.03.010 28380382 PMC12285277

[11] Mochel F, Knight MA, Tong WH, Hernandez D, Ayyad K, Taivassalo T, Andersen PM, Singleton A, Rouault TA, Fischbeck KH, Haller RG Splice mutation in the iron-sulfur cluster scaffold protein ISCU causes myopathy with exercise intolerance. Am J Hum Genet 2008;82:652–60. 10.1016/j.ajhg.2007.12.012 18304497 PMC2427212

[12] Olsson A, Lind L, Thornell LE, Holmberg M Myopathy with lactic acidosis is linked to chromosome 12q23.3-24.11 and caused by an intron mutation in the ISCU gene resulting in a splicing defect. Hum Mol Genet 2008;17:1666–72. 10.1093/hmg/ddn057 18296749

[13] Nordin A, Larsson E, Thornell LE, Holmberg M Tissue-specific splicing of ISCU results in a skeletal muscle phenotype in myopathy with lactic acidosis, while complete loss of ISCU results in early embryonic death in mice. Hum Genet 2011;129:371–8. 10.1007/s00439-010-0931-3 21165651

[14] Kollberg G, Tulinius M, Melberg A, Darin N, Andersen O, Holmgren D, Oldfors A, Holme E Clinical manifestation and a new ISCU mutation in iron-sulphur cluster deficiency myopathy. Brain 2009;132:2170–9. 10.1093/brain/awp152 19567699

[15] Sciacco M, Bonilla E Cytochemistry and immunocytochemistry of mitochondria in tissue sections. Methods Enzymol 1996;264:509–21. 8965723 10.1016/s0076-6879(96)64045-2

[16] Bugiani M, Invernizzi F, Alberio S, Briem E, Lamantea E, Carrara F, Moroni I, Farina L, Spada M, Donati MA, Uziel G, Zeviani M Clinical and molecular findings in children with complex I deficiency. Biochim Biophys Acta 2004;1659:136–47. 10.1016/j.bbabio.2004.09.006 15576045

[17] Uziel G, Garavaglia B, Di Donato S Carnitine stimulation of pyruvate dehydrogenase complex (PDHC) in isolated human skeletal muscle mitochondria. Muscle Nerve 1988;11:720–4. 10.1002/mus.880110708 3405240

[18] Stevens A Pigments and minerals : Bancroft JD, Stevens A, Theory and practice of histological techniques. Edinburgh: Churchill Livingstone, 1990:245–67.

[19] Legati A, Reyes A, Nasca A, Invernizzi F, Lamantea E, Tiranti V, Garavaglia B, Lamperti C, Ardissone A, Moroni I, Robinson A, Ghezzi D, Zeviani M New genes and pathomechanisms in mitochondrial disorders unraveled by NGS technologies. Biochim Biophys Acta 2016;1857:1326–35. 10.1016/j.bbabio.2016.02.022 26968897

[20] Biederbick A, Stehling O, Rösser R, Niggemeyer B, Nakai Y, Elsässer HP, Lill R Role of human mitochondrial Nfs1 in cytosolic iron-sulfur protein biogenesis and iron regulation. Mol Cell Biol 2006;26:5675–87. 10.1128/MCB.00112-06 16847322 PMC1592756

[21] Bonneaud N, Ozier-Kalogeropoulos O, Li GY, Labouesse M, Minvielle-Sebastia L, Lacroute F A family of low and high copy replicative, integrative and single-stranded S. cerevisiae/E. coli shuttle vectors. Yeast 1991;7:609–15. 10.1002/yea.320070609 1767589

[22] Sambrook J, Russel DW Molecular cloning: a laboratory manual. Cold Spring Harbor: Cold Spring Harbor Laboratory Press, 2001.

[23] Brachmann CB, Davies A, Cost GJ, Caputo E, Li J, Hieter P, Boeke JD Designer deletion strains derived from Saccharomyces cerevisiae S288C: a useful set of strains and plasmids for PCR-mediated gene disruption and other applications. Yeast 1998;14:115–32. 10.1002/(SICI)1097-0061(19980130)14:2<115::AID-YEA204>3.0.CO;2-2 9483801

[24] Gietz RD, Schiestl RH Quick and easy yeast transformation using the LiAc/SS carrier DNA/PEG method. Nat Protoc 2007;2:35–7. 10.1038/nprot.2007.14 17401335

[25] Ho SN, Hunt HD, Horton RM, Pullen JK, Pease LR Site-directed mutagenesis by overlap extension using the polymerase chain reaction. Gene 1989;77:51–9. 2744487 10.1016/0378-1119(89)90358-2

[26] Barrientos A, Fontanesi F, Díaz F Evaluation of the mitochondrial respiratory chain and oxidative phosphorylation system using polarography and spectrophotometric enzyme assays. Curr Protoc Hum Genet 2009;Chapter 19:Unit19.3 10.1002/0471142905.hg1903s63 PMC2771113 19806590

[27] Soto IC, Fontanesi F, Valledor M, Horn D, Singh R, Barrientos A Synthesis of cytochrome c oxidase subunit 1 is translationally downregulated in the absence of functional F1F0-ATP synthase. Biochim Biophys Acta 2009;1793:1776–86. 10.1016/j.bbamcr.2009.09.002 19735676 PMC2764804

[28] Patil VA, Fox JL, Gohil VM, Winge DR, Greenberg ML Loss of cardiolipin leads to perturbation of mitochondrial and cellular iron homeostasis. J Biol Chem 2013;288:1696–705. 10.1074/jbc.M112.428938 23192348 PMC3548480

[29] Molik S, Lill R, Mühlenhoff U Methods for studying iron metabolism in yeast mitochondria. Methods Cell Biol 2007;80:261–80. 10.1016/S0091-679X(06)80013-0 17445699

[30] Almeida T, Marques M, Mojzita D, Amorim MA, Silva RD, Almeida B, Rodrigues P, Ludovico P, Hohmann S, Moradas-Ferreira P, Côrte-Real M, Costa V Isc1p plays a key role in hydrogen peroxide resistance and chronological lifespan through modulation of iron levels and apoptosis. Mol Biol Cell 2008;19:865–76. 10.1091/mbc.E07-06-0604 18162582 PMC2262964

[31] Tamarit J, Irazusta V, Moreno-Cermeño A, Ros J Colorimetric assay for the quantitation of iron in yeast. Anal Biochem 2006;351:149–51. 10.1016/j.ab.2005.12.001 16403430

[32] Humphrey W, Dalke A, Schulten K VMD: visual molecular dynamics. J Mol Graph 1996;14:33–8. 10.1016/0263-7855(96)00018-5 8744570

[33] Guex N, Peitsch MC SWISS-MODEL and the Swiss-PdbViewer: an environment for comparative protein modeling. Electrophoresis 1997;18:2714–23. 10.1002/elps.1150181505 9504803

[34] Schilke B, Voisine C, Beinert H, Craig E Evidence for a conserved system for iron metabolism in the mitochondria of Saccharomyces cerevisiae. Proc Natl Acad Sci U S A 1999;96:10206–11. 10.1073/pnas.96.18.10206 10468587 PMC17867

[35] Garland SA, Hoff K, Vickery LE, Culotta VC Saccharomyces cerevisiae ISU1 and ISU2: members of a well-conserved gene family for iron-sulfur cluster assembly. J Mol Biol 1999;294:897–907. 10.1006/jmbi.1999.3294 10588895

[36] Mühlenhoff U, Gerber J, Richhardt N, Lill R Components involved in assembly and dislocation of iron-sulfur clusters on the scaffold protein Isu1p. Embo J 2003;22:4815–25. 10.1093/emboj/cdg446 12970193 PMC212715

[37] Gerber J, Neumann K, Prohl C, Mühlenhoff U, Lill R The yeast scaffold proteins Isu1p and Isu2p are required inside mitochondria for maturation of cytosolic Fe/S proteins. Mol Cell Biol 2004;24:4848–57. 10.1128/MCB.24.11.4848-4857.2004 15143178 PMC416415

[38] Shi R, Proteau A, Villarroya M, Moukadiri I, Zhang L, Trempe JF, Matte A, Armengod ME, Cygler M Structural basis for Fe-S cluster assembly and tRNA thiolation mediated by IscS protein-protein interactions. PLoS Biol 2010;8:e1000354 10.1371/journal.pbio.1000354 20404999 PMC2854127

[39] Cory SA, Van Vranken JG, Brignole EJ, Patra S, Winge DR, Drennan CL, Rutter J, Barondeau DP Structure of human Fe-S assembly subcomplex reveals unexpected cysteine desulfurase architecture and acyl-ACP-ISD11 interactions. Proc Natl Acad Sci U S A 2017;114:E5325–E5334. 10.1073/pnas.1702849114 28634302 PMC5502623

[40] Gakh O, Ranatunga W Smith DY4th, Ahlgren EC, Al-Karadaghi S, Thompson JR, Isaya G. Architecture of the Human Mitochondrial Iron-Sulfur Cluster Assembly Machinery. J. Biol. Chem 2016;291:21296–321. 27519411 10.1074/jbc.M116.738542 PMC5076535

[41] Stiban J, So M, Kaguni LS Iron-Sulfur Clusters in Mitochondrial Metabolism: Multifaceted Roles of a Simple Cofactor. Biochemistry 2016;81:1066–80. 10.1134/S0006297916100059 27908232

[42] Freibert SA, Goldberg AV, Hacker C, Molik S, Dean P, Williams TA, Nakjang S, Long S, Sendra K, Bill E, Heinz E, Hirt RP, Lucocq JM, Embley TM, Lill R Evolutionary conservation and in vitro reconstitution of microsporidian iron-sulfur cluster biosynthesis. Nat Commun 2017;8:13932 10.1038/ncomms13932 28051091 PMC5216125

[43] Saha PP, Kumar SK, Srivastava S, Sinha D, Pareek G, D’Silva P The presence of multiple cellular defects associated with a novel G50E iron-sulfur cluster scaffold protein (ISCU) mutation leads to development of mitochondrial myopathy. J Biol Chem 2014;289:10359–77. 10.1074/jbc.M113.526665 24573684 PMC4036159

[44] Sanaker PS, Toompuu M, Hogan VE, He L, Tzoulis C, Chrzanowska-Lightowlers ZM, Taylor RW, Bindoff LA Differences in RNA processing underlie the tissue specific phenotype of ISCU myopathy. Biochim Biophys Acta 2010;1802:539–44. 10.1016/j.bbadis.2010.02.010 20206689

[45] Crooks DR, Jeong SY, Tong WH, Ghosh MC, Olivierre H, Haller RG, Rouault TA Tissue specificity of a human mitochondrial disease: differentiation-enhanced mis-splicing of the Fe-S scaffold gene ISCU renders patient cells more sensitive to oxidative stress in ISCU myopathy. J Biol Chem 2012;287:40119–30. 10.1074/jbc.M112.418889 23035118 PMC3504726

[46] Holmes-Hampton GP, Crooks DR, Haller RG, Guo S, Freier SM, Monia BP, Rouault TA Use of antisense oligonucleotides to correct the splicing error in ISCU myopathy patient cell lines. Hum Mol Genet 2016;25:ddw338–5187. 10.1093/hmg/ddw338 PMC6078641 28007899

[47] Powers SK, Jackson MJ Exercise-induced oxidative stress: cellular mechanisms and impact on muscle force production. Physiol Rev 2008;88:1243–76. 10.1152/physrev.00031.2007 18923182 PMC2909187

[48] Dailey HA, Finnegan MG, Johnson MK Human ferrochelatase is an iron-sulfur protein. Biochemistry 1994;33:403–7. 10.1021/bi00168a003 8286370

[49] Sheftel AD, Wilbrecht C, Stehling O, Niggemeyer B, Elsässer HP, Mühlenhoff U, Lill R The human mitochondrial ISCA1, ISCA2, and IBA57 proteins are required for [4Fe-4S] protein maturation. Mol Biol Cell 2012;23:1157–66. 10.1091/mbc.E11-09-0772 22323289 PMC3315811

[50] Gelling C, Dawes IW, Richhardt N, Lill R, Mühlenhoff U Mitochondrial Iba57p is required for Fe/S cluster formation on aconitase and activation of radical SAM enzymes. Mol Cell Biol 2008;28:1851–61. 10.1128/MCB.01963-07 18086897 PMC2258791

[51] Nachbauer W, Boesch S, Reindl M, Eigentler A, Hufler K, Poewe W, Löscher W, Wanschitz J Skeletal muscle involvement in friedreich ataxia and potential effects of recombinant human erythropoietin administration on muscle regeneration and neovascularization. J Neuropathol Exp Neurol 2012;71:708–15. 10.1097/NEN.0b013e31825fed76 22805773

