# Supplementary material for: A novel de novo dominant mutation in ISCU associated with mitochondrial myopathy
Source: J Med Genet. 2017 Oct 27;54(12):815–24. doi: 10.1136/jmedgenet-2017-104822 (PMC5740555; doi:10.1136/jmedgenet-2017-104822)
Supplement: Supplementary material 1 [file jmedgenet-2017-104822supp001.pdf]

A novel *de novo* dominant mutation in *ISCU* associated with mitochondrial myopathy

Legati A, Reyes A, et al.

SUPPLEMENTARY DATA

Supplementary information about yeast studies

Yeast strains, media, cloning procedures and vectors. Yeast strains used in this work were W303-1B (MATα, *ade2-1 leu2-3,112 ura3-1 his3-22,15 trp1-1 can1-100*), and its isogenic strains *isu1::*hphMX4^R*isu2::*KanMX4 harboring plasmid pFL38*ISU1* (see below). Cells were cultured in yeast nitrogen base (YNB) medium [0.69% yeast nitrogen base without amino acids (Formedium™, UK)] supplemented with appropriate amino acids and bases for auxotrophy, except those required for plasmid maintenance or in YP medium (0,5% yeast extract (Formedium) and 1% peptone (Formedium). Carbon sources (Carlo Erba Reagents, Italy) were added as indicated in the text in liquid phase or after solidification with 20 g/l agar (ForMedium). Strains were incubated at 28°C. Minimal medium lacking $FeCl_3$ was obtained from Formedium. The *GAL1-10* promoter exchange strain *Gal-ISU1/isu2Δ* was depleted to critical protein levels by cultivation in SD medium for 16 h prior to analysis.

The yeast *ISU1* gene, including its upstream and downstream regulatory regions, was first PCR amplified using genomic DNA of strain W303-1B as template and ISU1BFw and ISU1HRv as primers (forward 5'-CCCCGGATCCGCTTCGTATTTGTCTTCCGTC-3' and reverse 5'-CCCCAAGCTTGCCAAAGGTCATGAGACTTGC-3'). The oligos were modified at 5' end and 3' end in order to insert restriction site for cloning in the centromeric plasmid pFL38 carrying the *URA3* marker [21]. The cloned fragment was sequenced to check the absence of mutations. Restriction-enzyme digestions, *Escherichia coli* transformation, and plasmid extractions were performed with standard methods [22].

Yeast strain construction and generation of mutant allele. Double deletion mutant *isu1*Δ *isu2*Δ was constructed by one step gene disruption in two steps. At first, the *isu1*::KanMX4 cassette was amplified from the genomic DNA of BY4741 *isu1Δ* [23] and inserted into W303-1B strain through high efficiency yeast transformation protocol [24]. The transformants were selected on YP supplemented with 200μg/ml geneticin and the correctness of disruption was confirmed by PCR. The KanMX4 marker was then replaced with Hyg^R marker obtained by digestion of pAG32 with *Hin*dIII and *Eco*RV. The *isu1*::KanMX4 strain was transformed with the Hyg^R cassette and the hygromycin resistant clones were selected. Subsequently, the pFL38 plasmid-borne *ISU1* was inserted into W303-1B *isu1*Δ to allow

cell viability and the resident *ISU2* was deleted by transforming *isu1*Δ/pFL38*ISU1* with the *isu2*::KanMX4 cassette amplified from genomic DNA of BY4741 *isu2*Δ. The transformants resistant to both antibiotics were selected and correct gene disruption was confirmed by PCR.

The conserved human glycine 96 residue, which is replaced by a valine in human *ISCU*, corresponds to glycine 97 in the yeast protein. The *isu1*^{G97V} mutant allele was obtained by site-direct mutagenesis using the overlap extension technique [25]. In the first set of PCR reactions, the *ISU1* region was obtained using the forward primer ISU1BFw and the following reverse mutagenic primer isu1G97VRv 5'-GAAGAGGAGGCAATGGCAGA**AAC**ACATCCAAAAGTTTTGAATTTG-3' where base changes are indicated in bold. The second *ISU1* region was obtained using the forward mutagenic primer isu1G97VFw, complementary to isu1G97VRv, and the reverse primer ISU1HRv. The final mutagenized product was obtained by using the overlapping PCR fragments as template with ISU1BFw and ISU1HRv as external primers. The product was then digested with *Bam*HI and *Hin*dIII and cloned in *Bam*HI-*Hin*dIII digested pFL39 centromeric plasmid [21]. The mutagenized insert was verified by sequencing and the pFL39/*isu1*^{G97V} mutant allele was transformed in the W303 *isu1*Δ*isu2*Δ/pFL38*ISU1*. Loss of pFL38/ *ISU1* was induced by growing the transformants on 5-fluoroorotic acid containing medium.

Supplementary Table S1: List of the antibodies used in this study

Antibody	Source
Rabbit a-ISCU	Lill laboratory
Rabbit a-FeCh	Kind Donation of T. And H. Dailey, Georgia, USA
Rabbit a-ACO2	Abgent (AP1936c)
Rabbit a-DLAT	Thermo Scientific (PA5-29043)
Rabbit a-Lipoic Acid	Calbiochem (437695)
Mouse a-NDUFA13	Abcam (6E1BH7)
Mouse a-NDUFB4	Abcam (17G3D9E12)
Mouse a-NDUFB6	Abcam (21C11BC11)
Mouse a-SDHB	Abcam (21A11AE7)
Rabbit a-UQCRFS1	Kind donation of H. Schägger and I. Wittig, Frankfurt, Germany
Rabbit a-COX2	H. Schägger and I. Wittig,
Rabbit a-F1b	H. Schägger and I. Wittig,
Rabbit a-IOP1	Lill laboratory
Rabbit a-POLD1	PTG Lab (15646-1-AP)
Rabbit a-NTHL1	Lill laboratory
Rabbit a-DPYD	SCBT (sc-50521)
Rabbit a-GPAT	Kind Donation of H. Puccio, IGBMC, France
Mouse a-IRP1	Kind Donation of R. Eisenstein, Wisconsin, USA
Mouse a-IRP2	SCBT (sc-33682)
Mouse a-tubulin	Sigma-Aldrich (clone DM1A)
Mouse a-actin	SCBT (sc-47778)
Mouse a-GAPDH	EMD Millipore (CB1001)

Figure S1.

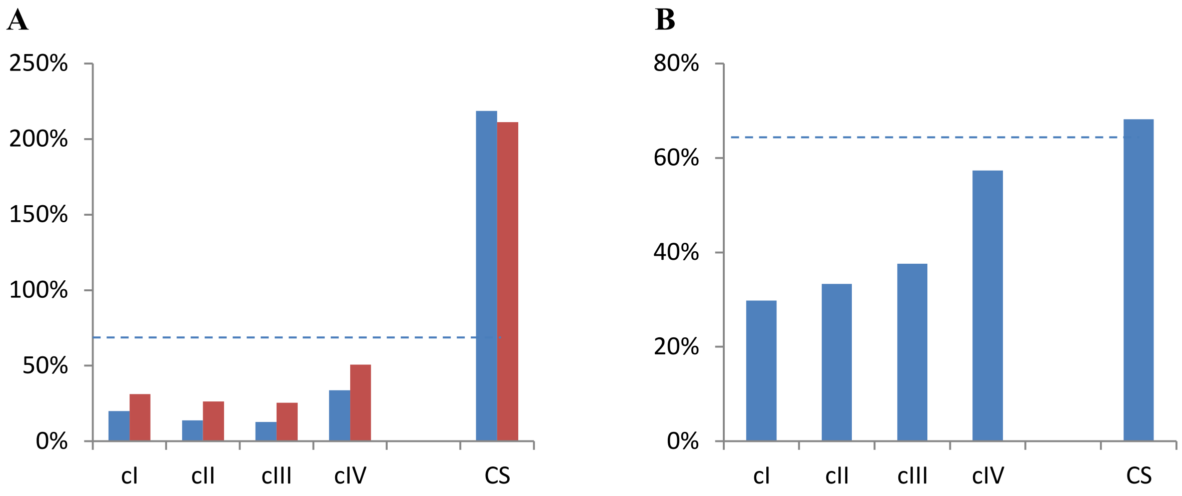

Supplementary Figure S1. Mitochondrial respiratory chain activities in patient's muscle

Activities of the respiratory chain complexes measured in patient's samples obtained from two different muscle biopsies, the first taken at 7 years of age (panel A; blue and red bars represent two independent measurements) and the second at 22 years (panel B). The activities were normalized for citrate synthase (CS) activity and reported as percentages of the mean control value (set as 100%). The dotted lines indicate the lower values in the control range.

Figure S2

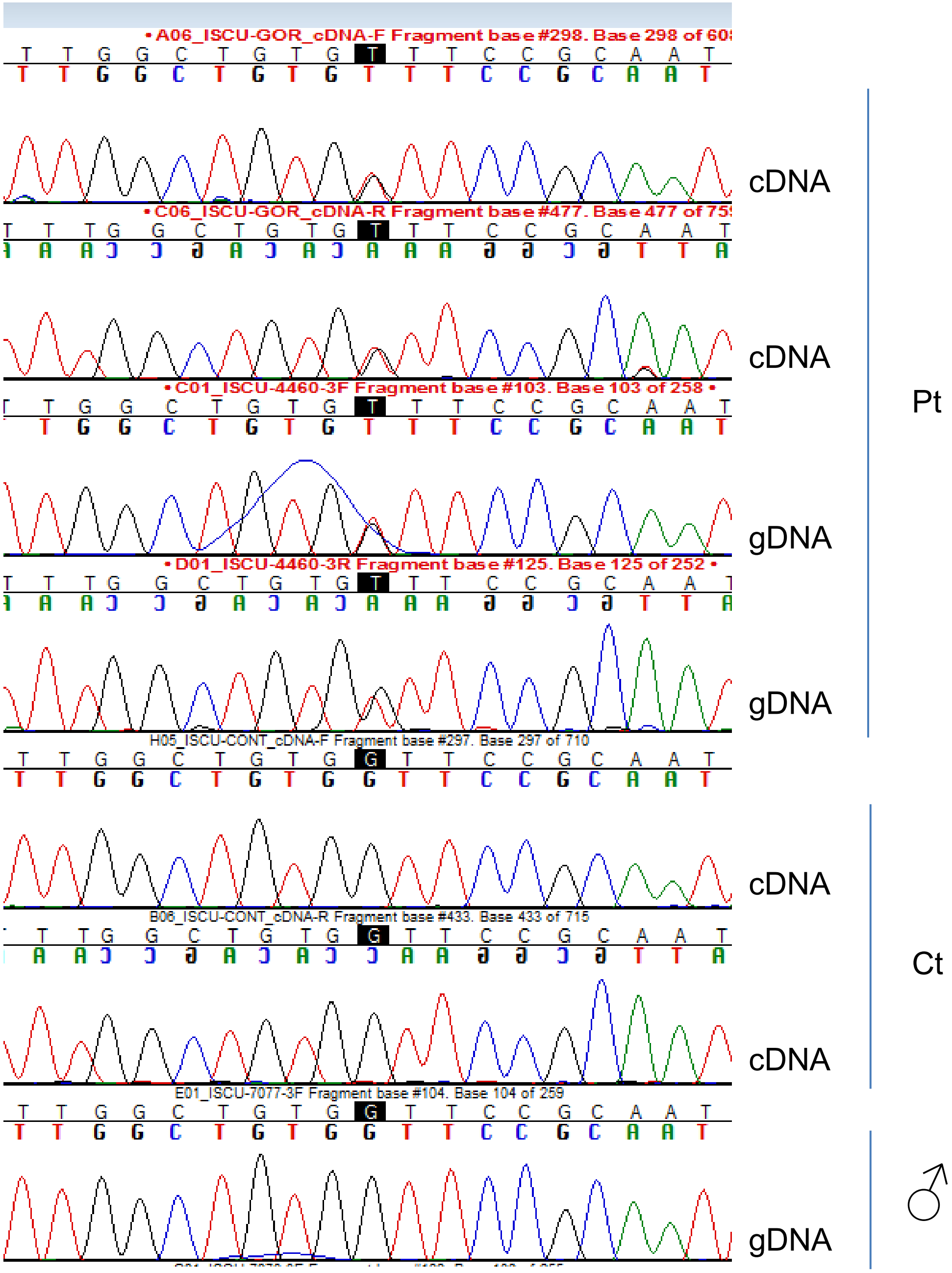

Supplementary Figure S2. Genetic analysis

Electropherograms of the genomic region (gDNA) and transcript (cDNA) harboring the *ISCU* variant c.287G>T, obtained from the patient (Pt), her father (♂) and a control subject (Ct).

Figure S3

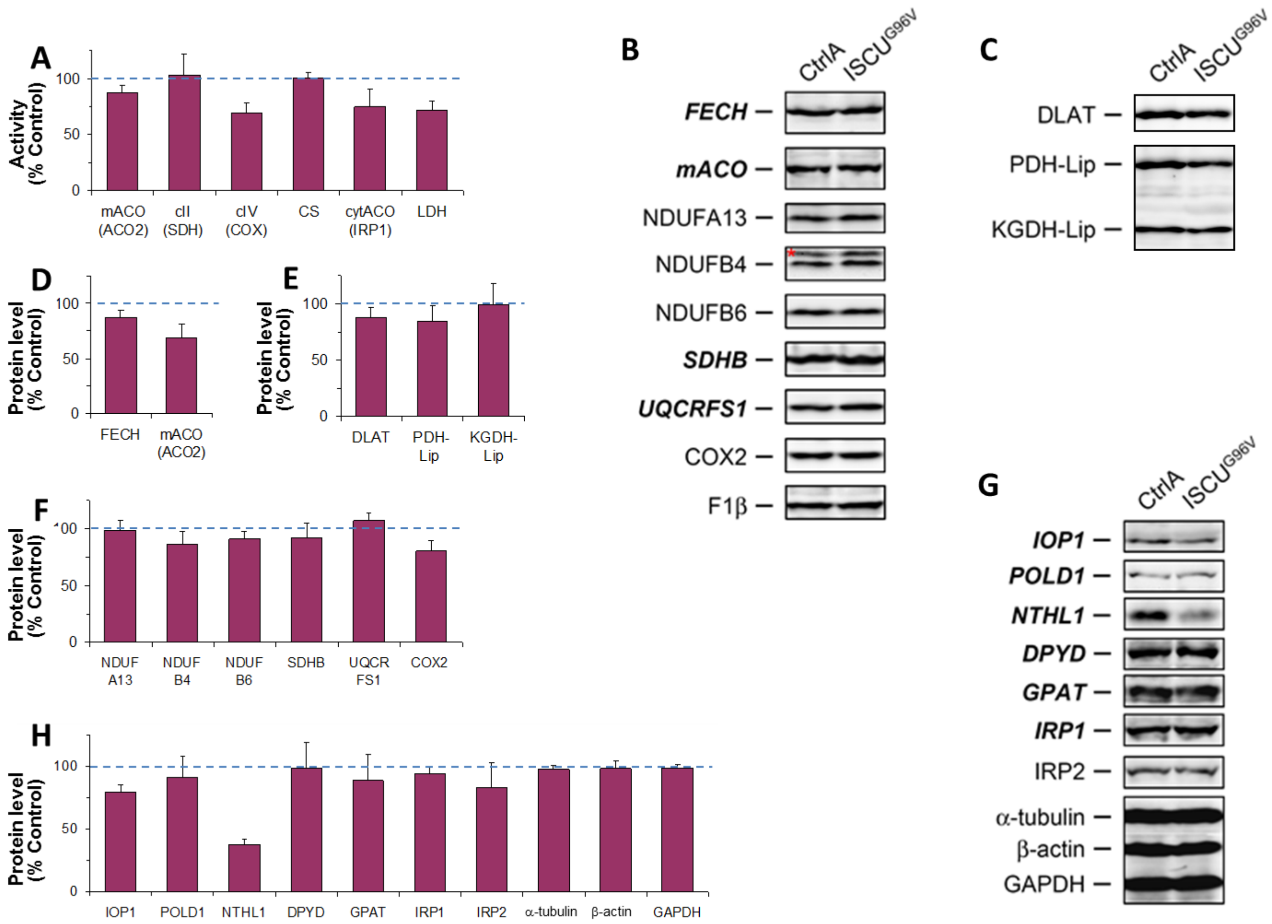

Supplementary Figure S3. Characterization of cultured patient fibroblasts.

Fibroblasts were treated as in Fig. 2D, and analysed for enzyme activities (A) and for steady-state protein levels (B, C, G; Fe-S proteins are highlighted in ***bold italics***). In bar graphs (A, D, E, F, H) values obtained from patient fibroblast samples were expressed relative to control cells (set as 100%). **A)** Mitochondrial fractions were analysed for enzymatic activities of succinate dehydrogenase (SDH, respiratory chain complex II, cII), cytochrome c oxidase (COX, respiratory chain complex IV, cIV), mitochondrial aconitase (mtACO, ACO2), and citrate synthase (CS). Cytosolic fractions were analysed for activities of cytosolic aconitase (cytACO, ACO1, IRP1) and lactate dehydrogenase (LDH). **B)** Representative immunoblot of mitochondrial Fe-S proteins (ferrochelatase, FECH; mitochondrial aconitase, mtACO) and MRC subunits (cI: NDUFA13, NDUFB4, NDUFB6; cII: SDHB; cIII: UQCRFS1/Rieske; cIV: COX2). **C**) Total cell lysates were analysed for LIAS activity by immunoblotting for covalently attached lipoate (Lip) on the E2 subunits of pyruvate dehydrogenase (PDH) and 2-ketoglutarate dehydrogenase (KGDH). PDH-E2 polypeptide (DLAT) was subsequently immunostained on the same blot and served as reference. **D, E, F)** Chemiluminescence signals of immunoblots as presented in panels B and C were quantified, and values obtained from patient fibroblast samples were expressed relative to control cells. **G)** Representative immunoblot of cytosolic Fe-S proteins (IOP1: CIA factor; POLD1: catalytic subunit of DNA polymerase δ; NTHL1: Nth-like DNA glycosylase 1; DPYD: dihydropyrimidine dehydrogenase; GPAT: glutamine phosphoribosylpyrophosphate amidotransferase; IRP1: iron regulatory protein 1 / ACO1) and reference polypeptides (IRP2: iron regulatory protein 2; α-tubulin; β-actin; GAPDH: glyceraldehyde 3-phosphate dehydrogenase). *non-specific immunoblot signal. **H)** Chemiluminescence signals of immunoblots as presented in panel G were quantified, and values obtained from patient fibroblast samples were expressed relative to control cells. Values are given as mean ± SD ($n \geq 7$).

Figure S4

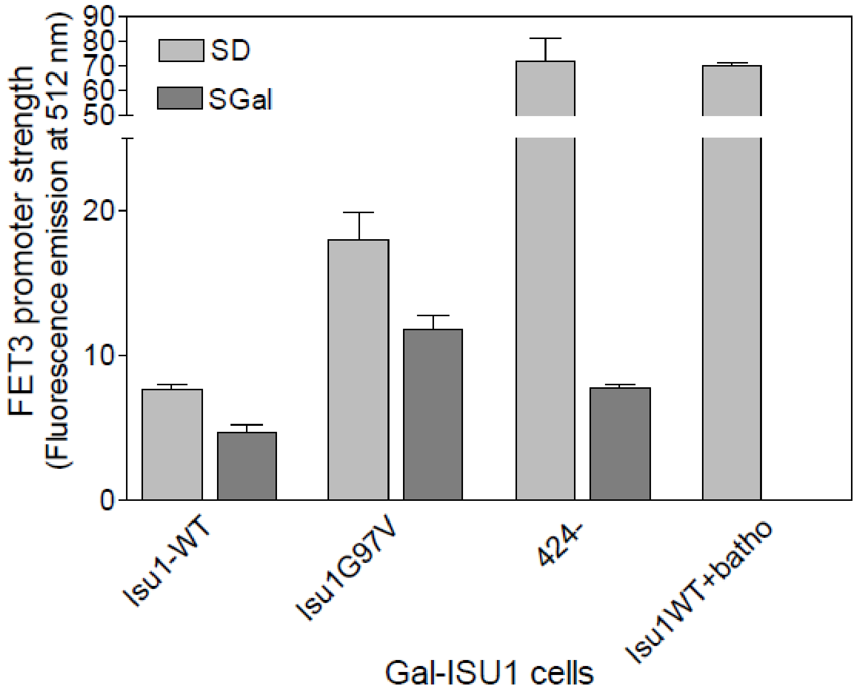

Supplementary Figure S4. Yeast analysis on iron uptake regulation

Gal-ISU1/*isu2*Δ cells were transformed with pFL39 vectors harbouring wild-type *ISU1* or *ISU1*-G97V and then with reporter plasmid p416FET3-GFP. Cells were grown in minimal (SD) medium for 16 h, diluted in fresh SD medium supplemented with 50 μM ferric ammonium citrate, and *FET3* promoter activities were determined by measuring the GFP-specific fluorescence emission of logarithmically growing cells. Error bars represent the SEM (n ≥ 4).
